# Commonly used clinical chemistry tests as mortality predictors: Results from two large cohort studies

**DOI:** 10.1371/journal.pone.0241558

**Published:** 2020-11-05

**Authors:** Lars Lind, Daniela Zanetti, Marieann Högman, Lars Sundman, Erik Ingelsson

**Affiliations:** 1 Department of Medical Sciences, Uppsala University, Uppsala, Sweden; 2 Department of Medicine, Division of Cardiovascular Medicine, Stanford University School of Medicine, Stanford, CA, United States of America; 3 Stanford Cardiovascular Institute, Stanford, CA, United States of America; 4 Stanford Diabetes Research Center, Stanford, CA, United States of America; 5 Department of Public Health Medicine, County Council of Gävleborg, Gävle, Sweden; 6 Department of Medical Sciences, Molecular Epidemiology and Science for Life Laboratory, Uppsala University, Uppsala, Sweden; University of Louisville, UNITED STATES

## Abstract

**Background:**

The normal ranges for clinical chemistry tests are usually defined by cut-offs given by the distribution in healthy individuals. This approach does however not indicate if individuals outside the normal range are more prone to disease.

**Methods:**

We studied the associations and risk prediction of 11 plasma and serum biomarkers with all-cause mortality in two population-based cohorts: a Swedish cohort (X69) initiated in 1969, and the UK Biobank (UKB) initiated in 2006–2010, with up to 48- and 9-years follow-up, respectively.

**Results:**

In X69 and in UKB, 18,529 and 425,264 individuals were investigated, respectively. During the follow-up time, 14,475 deaths occurred in X69 and 17,116 in UKB. All evaluated tests were associated with mortality in X69 (*P*<0.0001, except bilirubin P<0.005). For calcium, blood urea nitrogen, bilirubin, hematocrit, uric acid, and iron, U-shaped associations were seen (*P*<0.0001). For leukocyte count, gamma-glutamyl transferase, alkaline phosphatases and lactate dehydrogenase, linear positive associations were seen, while for albumin the association was negative. Similar associations were seen in UKB. Addition of all biomarkers to a model with classical risk factors improved mortality prediction (delta C-statistics: +0.009 in X69 and +0.023 in UKB, *P*<0.00001 in both cohorts).

**Conclusions:**

Commonly used clinical chemistry tests were associated with all-cause mortality both in the medium- and long-term perspective, and improved mortality prediction beyond classical risk factors. Since both linear and U-shaped relationships were found, we propose to define the normal range of a clinical chemistry test based on its association with mortality, rather than from the distribution.

## Introduction

In the late sixties, the first multi-analyzers for delivering results of multiple commonly used clinical chemistry tests were manufactured. Since many decisions in clinical medicine are based on such common clinical chemistry tests, this was an important step in optimization of patient care.

The most commonly used procedure to define normal range for such clinical chemistry measurements is to measure the analyte of interest in a population of healthy individuals and then define the upper and lower normal limit as the 2.5^th^ and 97.5^th^ percentile. This approach has been denoted demographic reference intervals (DRI), which do not take into consideration whether values above or below those limits are associated with a poor health or not. An alternative way to define the normal range is to use levels of a clinical chemistry test that are associated with certain health outcomes, so called risk-based decision-limits (RBDL). This approach has been applied for certain analytes, especially in later years, such as troponins for acute myocardial infarction [1], NT-proBNP for heart failure [2], or lipid levels for prediction of cardiovascular risk [3].

Very few investigations have been carried out to investigate whether risk-based decision-limits could be applied for the most commonly analyzed clinical chemistry tests (rather than demographic reference intervals as currently are used). One prior study investigated five such common biomarkers: blood urea nitrogen (BUN), serum creatinine, sodium, potassium and chloride, as predictors of all-cause mortality during a 1-year follow-up in almost 40,000 hospitalized patients. They found U-shaped relationships for the four latter analytes, and could suggest risk-based decision-limits instead of demographic reference intervals for these five laboratory tests [4].

In 1969, a general health screening survey (X69) was conducted in the City of Gävle, Sweden, in which 18,529 adults participated (55% women). One of the early versions of a clinical chemistry multi-analyzer was used to measure 11 commonly used clinical chemistry tests (calcium, blood urea nitrogen [BUN], bilirubin, albumin, hematocrit, leukocyte count, uric acid, iron, gamma-glutamyl transferase [GGT], alkaline phosphatases [ALP] and lactate dehydrogenase [LD]) in that health screening project. Since we have been able to follow mortality of the X69 cohort for almost five decades, we have an outstanding opportunity to evaluate the association between these 11 biomarkers and all-cause mortality, both in the medium- and long-term perspective, to see if the method to define the normal ranges based on DRI is appropriate in relation to mortality, or if an RBDL approach would be more valid. To increase the validity of our results, we aimed to replicate our findings in the UK Biobank (UKB; N = 425,264). As a secondary aim, we also evaluated if these clinical chemistry tests could improve mortality prediction above traditional risk factors for mortality.

## Methods

### X69

In 1969, all citizens above 24 years of age in one part of the city of Gävle, Sweden, were invited to a health screening survey. The participation rate was 77%, resulting in 18,543 individuals (8,416 men and 10,127 women) being screened. The clinical chemistry tests failed in 14 individuals, so 18,529 participants were eligible for the present investigation.

A venous blood sample was drawn after at least 4h of fasting, mostly in the afternoon. These samples were analyzed in a Technicon Autoanalyzer SMAC 12/60 (Mark Technicon, USA) for measurements of the 11 clinical chemistry tests included in the study (calcium, BUN, bilirubin, albumin, hematocrit, leukocyte count, uric acid, iron, GGT, ALP and LD). In addition, blood pressure was measured in the supine position after 10 min of rest. BMI was defined as weight divided by the square of height. Smoking status was defined as current smoking.

Date and cause of mortality was extracted from the Swedish Cause of Death Register with no loss of follow-up due to the universal Swedish national identification numbers. The study was approved by the Ethics Committee of Uppsala University the 9^th^ of August 2017, Dnr 2017/286.

### UK Biobank (UKB)

The UKB is a longitudinal cohort study of >500,000 individuals aged 40 to 69 years initiated in the United Kingdom in 2006–2010 [5]. We used data collected at the UKB assessment centers at baseline, combined with information collected in the national death register. Follow-up started at the baseline examination, and ended on January 31, 2018 in England and Wales, and November 30, 2016 in Scotland.

Analyses of serum biomarkers utilized ten immunoassay analyzers (six DiaSorin Liaison XL and four Beckman Coulter DXI 800) and four clinical chemistry analyzers (two Beckman Coulter AU5800 and two Siemens Advia 1800). Iron and LD were not measured in the UKB, while the other nine biomarkers were assessed. Fasting status was defined as ≥ 8 (fasting) or <8 (non-fasting) hours since last meal. Body mass index and blood pressure were measured at baseline using standard methods. Smoking status was recoded to three groups: never, previous or current. In the present study, we included the 425,264 UKB participants with biomarker measurements passing QC. Details of all measurements can be found in the UKB Data Showcase (http://biobank.ctsu.ox.ac.uk/crystal/). The UKB study was approved by the North West Multi-Centre Research Ethics Committee and all participants provided written informed consent.

### Statistical methods

First, in X69, we calculated Pearson´s correlation coefficient for the pairwise comparison between the 11 biomarkers. The biomarkers were first inverse rank normalized in order to assure normal distributions. The results are visualized as a heat map and a principal component (PCA) plot.

Second, Cox proportional hazards analyses were performed to evaluate the associations of traditional risk factors (age, sex, glucose, systolic blood pressure, total cholesterol, BMI, and smoking) with all-cause mortality. In this model, log natural-transformed values of glucose were used due to its skewed distribution. The proportional hazard assumption was evaluated by visual inspection of Kaplan-Maier curves where the biomarkers were split into two groups by the median. Additionally, for biomarkers with evidence of non-linearity, tertiles were used in the assessment of proportional hazards. The only variable with evidence of non-proportional hazards was bilirubin, where this occurred after 30 years of follow-up in X69. Hence, for this variable, only the first 20 years of follow-up were used in all downstream analyses.

Third, Cox proportional hazards analyses were used to evaluate associations between the biochemical variables and all-cause mortality. In the primary analysis, the different biomarkers were evaluated one by one in separate models adjusting for age, sex, glucose (along with fasting status in UKB), systolic blood pressure, total cholesterol, BMI, and smoking as potential confounders. In these analyses, the biochemical variables were modeled using cubic restricted spline functions with 3 knots (10^th^, 50^th^ and 90^th^ percentile) to allow for potential non-monotonic and other non-linear relationships. The results are presented as hazard ratios for the 1^st^, 5^th^, 10^th^, 25^th^, 50^th^, 75^th^, 90^th^, 95^th^ and 99^th^ percentile of the distribution for the biochemical variables. If a U-shaped relationship was observed with mortality, the median was set at the referent value. If linear relationships were seen, the 1^st^ percentile was used as reference.

In a secondary analysis, to match the length of follow-up in X69 with the UKB, the same models were performed using only the first 10 years of follow-up in X69. To address our secondary aim, using this 10-year follow-up period, we also calculated C-statistics (corresponding to the area under the receiver operator curve) by logistic regression. We started with a variable selection in X69 (using spline functions for the 9 biochemical variables that were available in UKB, plus age and sex) by use of lasso and the split sample technique (the model discovery step). The model with the lowest error was selected. We then examined the predictive capability of the classical risk factors and compared that with a model in which the spline functions of all biomarkers selected by lasso were added.

The same model was then evaluated in UKB as the validation step.

Cox regressions analyses were conducted with STATA14 (Stata Inc, College Station, TX, USA) for X69 and the R package *rms* (version 5.1–2) for the UKB.

## Results

### Relationships between biomarkers

**Fig 1:**
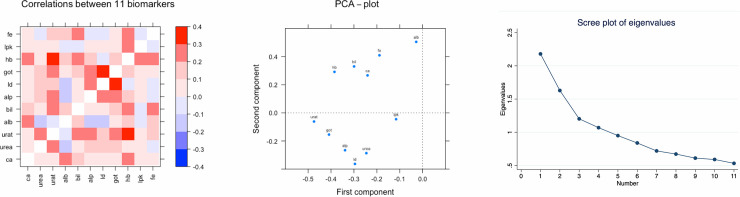
Relationships between the 11 biomarkers measured in the X69 sample given as correlation coefficients in a heat map and in a principal component (PCA) plot. Ca = calcium, urat = uric acid, alb = albumin, bil = bilirubin, alp = alkaine phosphatase, ld = lactate dehydrogenase, got = gamma glutamate transferase, hb = hematocrit, lpk = leukocyte count, fe = iron.

As could be seen in **Fig 1**, the relationships between the 11 biomarkers measured in the X69 sample showed correlation coefficients ranging from -0.12 (albumin vs ALP) to 0.34 (GOT vs LD). The PCA plot for the first two components disclosed two clusters. One with GOT, LD, ALP, urate and urea, and another with hematocrit, iron, calcium and bilirubin. The scree plot part of the figure shows the eigenvalues for the identified components. The first component captures 0.20 of the variation and the second components captures 0.15.

### Associations of traditional risk factors and all-cause mortality

**Fig 2:**
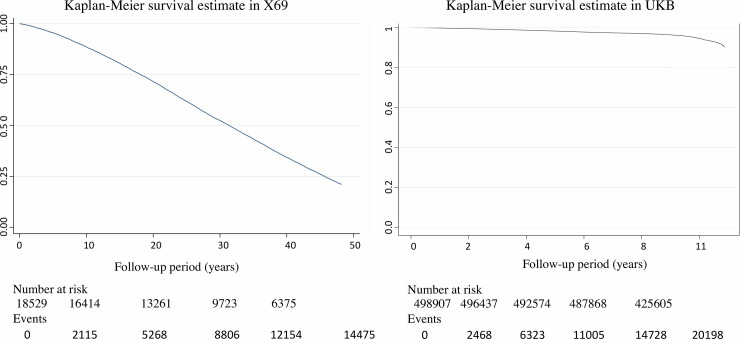
Kaplan-Meier survival curves for the follow-up period in the two samples.

Baseline characteristics of X69 and UKB are shown in **Table 1**. During a median follow-up time of 31.3 years (min, 0.01; max, 48.2 years), 14,475 deaths occurred in X69 during 559,878 person-years at risk; mortality rate of 25.8/1,000 years at risk. In UKB, the median follow-up was 9.0 years (min, 0.01; max, 12.1 years) during which 17,116 deaths occurred (3,777,934 person-years at risk; mortality rate 4.5/1,000 years at risk). The mortality trends over time in the two samples are graphically shown by Kaplan-Meier curves in **Fig 2**.

**Table 1:** Distributions of traditional risk factors and their associations with all-cause mortality in X69 and UK Biobank.

	X69				UK Biobank			
	Mean (SD) or %	HR	95% CI	P-value	Mean (SD) or %	HR	95% CI	P-value
Age, years	49.0 (14)	1.004	1.002–1.005	2.4E-07	56.5 (8.1)	1.098	1.096–1.101	<2E-16
Female sex, %	55	0.66	0.63–0.68	1.0E-120	54	0.548	0.534–0.565	<2E-16
BMI, kg/m^2^	24.0 (3.5)	1.01	1.010–1.020	2.1E-09	27.4 (4.8)	1.159	1.144–1.174	<2E-16
Glucose, mmol/l	5.8 (1.1)	1.74	1.52–2.00	9.3E-16	5.0 (0.7)	1.380	1.356–1.404	<2E-16
Systolic blood pressure, mmHg	139 (23)	1.008	1.007–1.009	1.6E-89	140 (20)	1.246	1.230–1.262	<2E-16
Smoking, %	38	1.63	1.57–1.69	7.0E-157	11	1.696	1.665–1.728	<2E-16
Total cholesterol, mmol/l	6.8 (1.3)	1.03	1.02–1.05	2.2E-08	5.7 (1.1)	0.806	0.796–0.817	<2E-16

Means (standard deviations [SD]) or proportions for traditional risk factors and their associations with all-cause mortality in X69 and UK Biobank using Cox regressions. The hazard ratio (HR) is for a 1-unit increase for continuous variables. For sex, the HR is given for women compared to men. Glucose is given on a ln-scale.

Abbreviations: BMI, body mass index; CI, confidence interval.

All traditional risk factors were associated with all-cause mortality in both study samples (**Table 1**).

### Associations of biomarkers with all-cause mortality in X69

**Fig 3:**
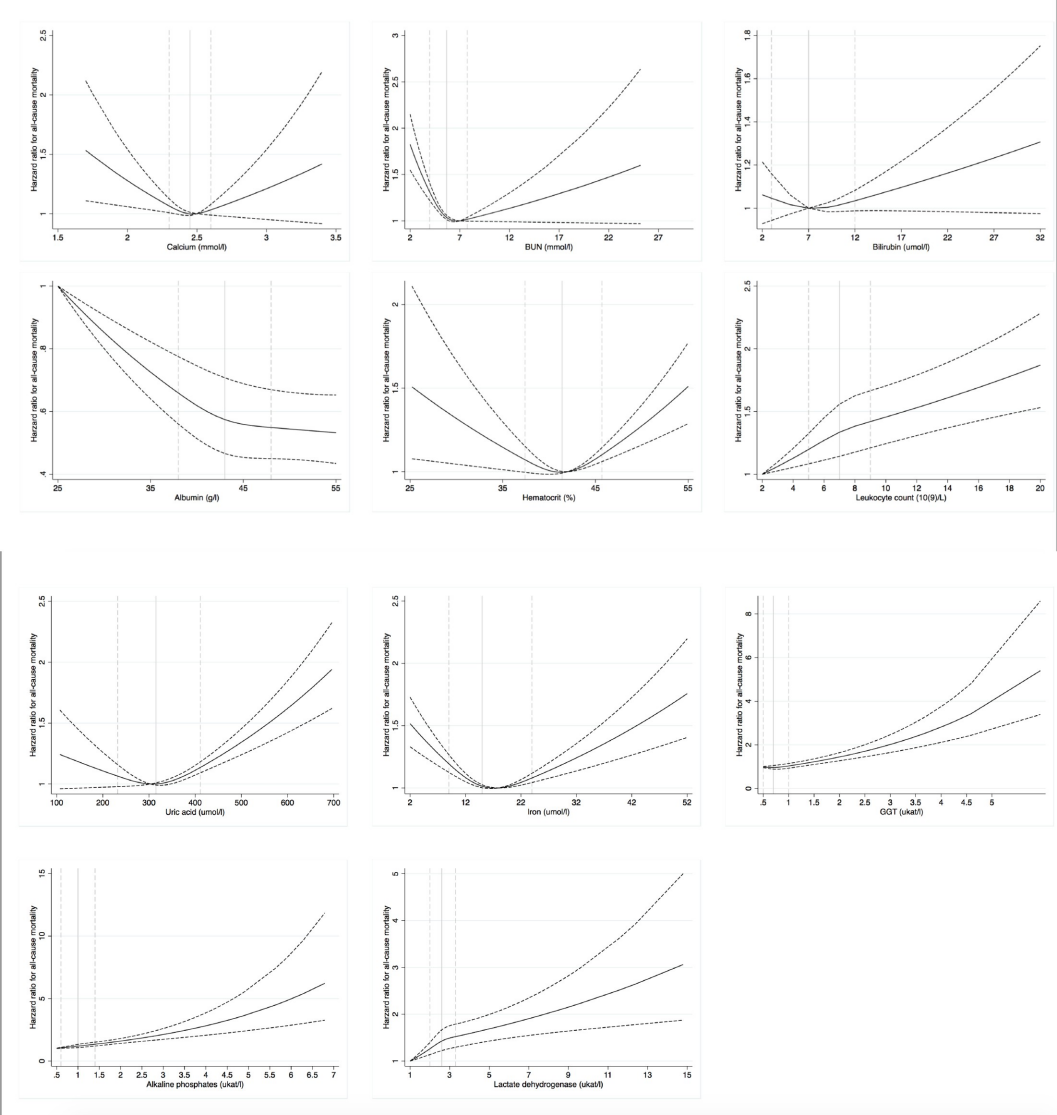
Associations between 11 clinical biomarkers and all-cause mortality over 48 years in the X69 cohort. The 10^th^ percentile (dashed), median (solid) and 90^th^ percentile (dashed) of the biomarker levels are indicated by vertical lines. A restricted cubic spline function with three knots were used for the calculations with hazard ratio given as a solid line, upper and lower 95% confidence intervals as dashed lines. All relationships showed p<0.0001 vs mortality, except bilirubin (p = 0.0034).

Highly significant associations for 10 of the evaluated biomarkers (all except bilirubin) with all-cause mortality were observed in X69 (*P*<0.0001; **Fig 3 and Table 2**) when they were evaluated one by one adjusting for classical risk factors (age, sex, glucose, systolic blood pressure, BMI, total cholesterol and smoking). For calcium, BUN, hematocrit, uric acid and iron, U-shaped associations were seen (*P*<0.0001 for non–linearity). In contrast, for leukocyte count, GGT, ALP and LD, linear positive associations were seen, while for albumin the association was negative. For bilirubin, the proportionality of hazards assumption only held for the first 20 years of follow-up. During that period, the curvilinear spline function was significantly related to mortality (*P* = 0.0034), but neither higher nor lower levels than the median were significantly related to mortality when analyzed as categorical variables.

**Table 2:** Associations between the 1^st^, 5^th^, 10^th^, 25^th^, 50^th^, 75^th^, 90^th^, 95^th^ and 99^th^ percentile of the distribution of 11 clinical chemistry tests and all-cause mortality in the X69 sample and for 9 of these tests in the UK Biobank.

	X69				UK Biobank			
	Percentile value	Hazard ratio	95% CI lower limit	95% CI upper limit	Percentile value	Hazard ratio	95% CI lower limit	95% CI upper limit
**Calcium (mmol/l)**	2.2	1.13	1.03	1.25	2.17	1.49	1.41	1.58
	2.3	1.07	1.01	1.13	2.24	1.29	1.25	1.34
	2.3	1.07	1.01	1.13	2.27	1.21	1.17	1.24
	2.4	1.01	0.99	1.03	2.32	1.08	1.07	1.09
	2.45	Referent			2.38	Referent		
	2.5	1.00	1.00	1.00	2.44	1.01	1.00	1.02
	2.6	1.03	0.99	1.08	2.50	1.07	1.04	1.10
	2.65	1.05	0.98	1.13	2.54	1.12	1.08	1.16
	2.75	1.10	0.97	1.23	2.63	1.25	1.17	1.33
**Blood urea nitrogen (BUN) (mmol/l)**	2.89	1.57	1.39	1.78	2.83	2.03	1.92	2.15
	3.53	1.41	1.29	1.55	3.49	1.63	1.56	1.69
	3.96	1.31	1.22	1.41	3.85	1.44	1.40	1.49
	4.75	1.16	1.11	1.21	4.49	1.17	1.16	1.19
	5.68	Referent			5.26	Referent		
	6.68	1.02	1.00	1.04	6.14	1.02	1.01	1.03
	7.75	1.02	1.00	1.06	7.07	1.20	1.18	1.23
	8.46	1.04	1.00	1.08	7.72	1.37	1.34	1.41
	10.0	1.08	0.99	1.17	9.25	1.87	1.78	1.97
**Bilirubin (umo/l)**	2	1.06	0.93	1.21	4	1.34	1.27	1.40
	3	1.05	0.94	1.16	5	1.25	1.21	1.30
	3	1.05	0.94	1.16	5	1.20	1.16	1.24
	5	1.02	0.97	1.06	6	1.11	1.09	1.12
	7	Referent			8	Referent		
	9	1.00	0.98	1.02	10	0.94	0.93	0.95
	12	1.03	0.99	1.08	13	0.96	0.94	0.98
	15	1.07	0.99	1.08	17	1.00	0.96	1.05
	21	1.15	0.98	1.34	24	1.10	1.01	1.19
**Albumin (g/l)**	34	Referent			39	2.31	2.20	2.43
	37	0.91	0.87	0.94	40	1.71	1.66	1.77
	38	0.88	0.84	0.92	41	1.48	1.45	1.52
	40	0.83	0.77	0.89	43	1.18	1.17	1.19
	43	0.77	0.70	0.84	45	Referent		
	45	0.75	0.68	0.82	46	0.97	0.96	0.99
	48	0.73	0.66	0.81	48	1.02	0.99	1.06
	49	0.73	0.66	0.81	49	1.07	1.02	1.12
	52	0.72	0.64	0.81	51	1.15	1.07	1.24
**Hematocrit (%)**	33.7	1.18	1.03	1.35	32.8	2.35	2.22	2.48
	36.2	1.10	1.02	1.19	35.5	1.72	1.66	1.78
	37.4	1.06	1.01	1.12	36.7	1.50	1.46	1.54
	39.3	1.01	1.00	1.03	38.7	1.20	1.18	1.21
	41.4	Referent			41.0	Referent		
	43.5	1.02	0.99	1.05	43.5	0.96	0.95	0.97
	45.7	1.09	1.04	1.15	45.6	1.00	0.97	1.03
	46.9	1.14	1.07	1.21	46.9	1.03	0.98	1.07
	49.3	1.24	1.13	1.35	49.2	1.09	1.02	1.16
**Leukocyte count (10**^**9**^**/l)**	4	Referent			4	1.11	1.04	1.18
	5	1.06	1.03	1.10	4	1.07	1.02	1.12
	5	1.06	1.03	1.10	5	1.04	1.01	1.08
	6	1.13	1.05	1.20	6	1.01	0.99	1.02
	7	1.18	1.08	1.29	7	Referent		
	8	1.23	1.12	1.35	8	1.11	1.10	1.12
	9	1.26	1.15	1.38	9	1.33	1.30	1.36
	10	1.29	1.18	1.42	10	1.53	1.49	1.58
	12	1.36	1.23	1.50	12	2.10	1.99	2.22
**Uric acid (umol/l)**	173	1.14	0.96	1.36	154	1.28	1.19	1.37
	208	1.10	0.97	1.24	189	1.19	1.13	1.25
	232	1.06	0.97	1.17	210	1.14	1.09	1.18
	268	1.02	0.98	1.07	250	1.05	1.03	1.07
	315	Referent			303	Referent		
	363	1.04	1.02	1.06	360	1.07	1.06	1.08
	411	1.14	1.10	1.18	415	1.23	1.19	1.26
	446	1.21	1.15	1.28	450	1.34	1.30	1.40
	524	1.40	1.28	1.54	519	1.62	1.53	1.72
**Iron (umol/l)**	5	1.42	1.29	1.57	N/A			
	7	1.32	1.22	1.43				
	9	1.22	1.15	1.30				
	11	1.13	1.09	1.18				
	15	Referent						
	19	1.01	1.00	1.01				
	24	1.08	1.05	1.12				
	27	1.15	1.09	1.21				
	35	1.33	1.19	1.48				
**Gamma glutamyl transferase (GGT) (ukat/l in X69 and U/l in UKB)**	0.42	Referent			9.4	Referent		
	0.49	0.99	0.97	1.01	10.2	1.01	1.00	1.01
	0.54	0.98	0.95	1.02	12.7	1.02	1.01	1.03
	0.61	0.97	0.92	1.04	14.5	1.03	1.01	1.04
	0.71	0.97	0.89	1.06	18.5	1.05	1.03	1.08
	0.84	0.99	0.89	1.09	41.0	1.21	1.14	1.29
	1.00	1.04	0.94	1.15	66.9	1.46	1.37	1.56
	1.15	1.09	0.99	1.21	94.8	1.80	1.70	1.91
	1.67	1.30	1.16	1.45	125.3	2.27	2.12	2.42
**Alkaline phophatases (ALP) (ukat/l in X69 and U/l in UKB)**	0.44	Referent			37.60	Referent		
	0.58	1.05	1.02	1.08	41.40	1.01	1.00	1.02
	0.65	1.07	1.02	1.12	51.30	1.03	1.00	1.06
	0.81	1.13	1.04	1.22	56.90	1.04	1.01	1.08
	1.00	1.20	1.08	1.34	67.20	1.07	1.01	1.13
	1.23	1.29	1.14	1.45	95.90	1.29	1.20	1.40
	1.48	1.38	1.23	1.55	113.00	1.57	1.46	1.70
	1.66	1.45	1.30	1.63	125.50	1.83	1.70	1.97
	2.14	1.67	1.46	1.89	155.70	2.63	2.44	2.83
**Lactate dehydrogenase (LD) (ukat/l)**	1	Referent			N/A			
	3	1.49	1.27	1.76				
	4	1.59	1.35	1.86				
	5	1.69	1.43	1.99				
	7	1.91	1.55	2.35				
	9	2.15	1.64	2.82				
	11	2.43	1.72	3.42				
	13	2.74	1.80	4.18				
	15	3.10	1.88	5.11				

The hazard ratios (HR) and the corresponding 95% confidence intervals vs all-cause mortality during the follow-up period (48 years in X69 and 9 years in UK Biobank) are given for the 1^st^, 5^th^, 10^th^, 25^th^, 50^th^, 75^th^, 90^th^, 95^th^ and 99^th^ percentile of the distribution. If a U-shaped relationship was observed, the median was set at the referent value. If monotonic relationship was seen, the 1^st^ percentile was set as the referent.

In secondary analyses, including only the first 10 years of follow-up in X69, 2,113 deaths occurred during 176,215 person-years at risk. Essentially identical observations were made with this shorter follow-up time, with highly significant relationships for all evaluated tests, except bilirubin.

### Replication in UK Biobank

**Fig 4:**
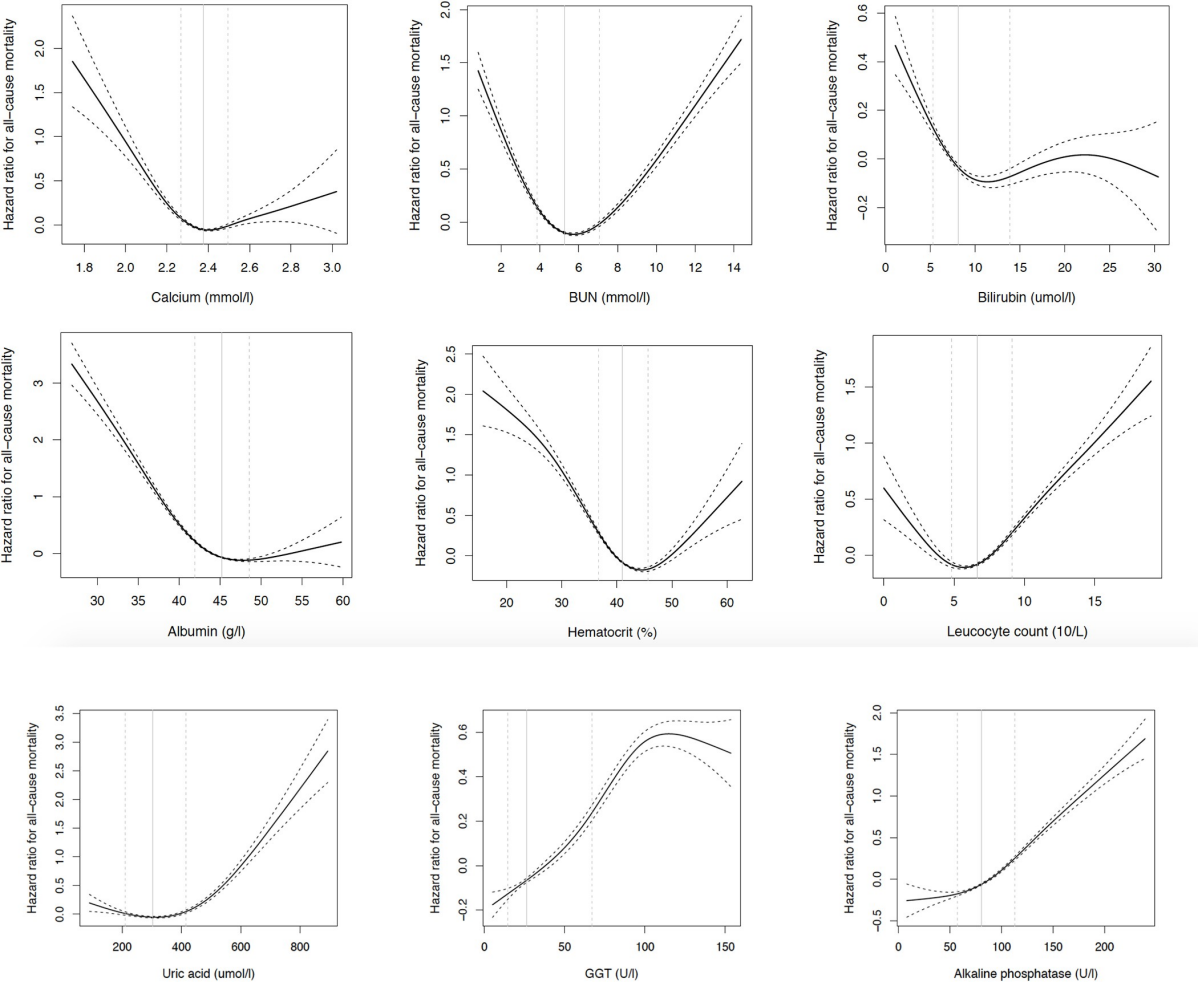
Relationships between 9 clinical chemistry tests and all-cause mortality over 9 years in the UK Biobank sample. The 10^th^ percentile (dashed), median (solid) and 90^th^ percentile (dashed) of the biomarkers are indicated by vertical lines. A restricted cubic spline function with three knots were used for the calculations with hazard ratio given as a solid line, upper and lower 95% confidence intervals as dashed lines.

Associations for the nine biomarkers that were available in UKB replicated with similarly shaped (and highly significant) associations as in X69, except that leukocyte count and albumin showed a U-shape relation with mortality (rather than linear as in X69). Also, a clearly elevated mortality risk was seen at low levels of bilirubin. The increased risk of having high levels of BUN and GGT, or low levels of leukocytes and uric acid were more exaggerated in UKB (**Fig 4 and Table 2)**.

### Using the biomarkers to improve mortality prediction

During the first 10 years of the follow-up in X69, the lasso procedure selected all 9 variables included in UKB in the model with the lowest error. The C-statistics of a model including all traditional risk factors for all-cause mortality was 0.855 (95% CI, 0.846–0.864). Addition of the 11 biochemical variables significantly increased the C-statistics to 0.862 (95% CI 0.854–0.871; delta C-statistics, +0.009; *P*<0.00001).

In UKB, a model with the classical risk factors resulted in a C-statistics of 0.733 (95% CI, 0.727–0.739). Including the same nine biochemical variables as in the model selected in X69 resulted in a substantial increase of C-statistics to 0.756 (95% CI, 0.750–0.762; delta C-statistics, +0.023; *P*<10^−15^).

## Discussion

### Principal findings

Using two large cohorts, all evaluated clinical chemistry tests were strongly associated with mortality during the follow-up period in a consistent manner. For calcium, BUN, hematocrit, uric acid, bilirubin and iron, U-shaped associations were seen. For leukocyte count, GGT, ALP and LD the relationships were positive, while for albumin, a negative association was observed. It should therefore be obvious that a definition of the normal range using the extremes of the distribution in healthy individuals is not appropriate from a public health perspective, at least not if future disease and mortality is the purpose of testing. We propose that normal values rather should be defined by associations with health outcomes, and that all-cause mortality is such an outcome of ultimate importance. Further, we demonstrate that the addition of these commonly used clinical chemistry tests to a model including classical risk factors substantially improved prediction of all-cause mortality in both X69 and UK Biobank.

### Comparison with the literature

Apart from one prior study evaluating mortality risk based on five different commonly used clinical chemistry tests in hospitalized patients [4], we are only aware of such analyses for single biomarkers, and most often in patient samples, not in the general population.

High serum calcium levels are mainly seen in primary hyperparathyroidism (HPT) and in certain cancers. In both cases, high serum calcium levels have been linked to increased mortality [6, 7]. A high calcium level has also been linked to increased mortality in the general male population [8, 9]. Hypocalcemia is commonly found in severe illness, such as sepsis or major trauma. In the case of hypocalcemia, low levels of calcium have been associated with increased mortality risk in the emergency department [10], as well in the general hospital population [11]. Thus, the U-shaped curve that we report for the relationship between calcium levels and mortality in the general population is not a surprising finding, and perfectly in line with prior literature.

High levels of BUN have been associated with increased mortality in several patient groups, such as cancer [12] and heart failure [13, 14]. High BUN at hospital discharge predicts mortality also in unselected patients [15], while a separate study did not observe an association of low BUN levels with post-discharge mortality [4]. The latter observation contrasts with our findings of a U-shaped association with more prominent risk increases for low BUN levels. We hypothesize that low BUN levels could be a marker of a poor nutritional intake that could affect mortality risk.

The bilirubin association was strong in UK Biobank with an increased mortality risk associated with both high and low bilirubin levels, while the association in X69 was weaker (although the global test was significant). An association of lower bilirubin levels with mortality fits well with the suggested cardiovascular-protective actions of bilirubin. As reviewed by Bulmer and colleagues [16], bilirubin within the normal range is associated with a reduction in oxidative lipid and protein modifications, as well as a reduced platelet activation. In addition, it has been noted that individuals with Gilbert´s syndrome, a state of unconjugated hyperbilirubinemia, are protected from cardiovascular disease and associated mortality [17]. On the other hand, in other conditions in which conjugated hyperbilirubinemia is common, like in heart failure, high bilirubin levels are associated with an increased mortality [18, 19]. Further, high levels of other liver function tests, such as aspartate transaminase, GGT and ALP, have been linked to increased mortality in heart failure patients [19]; but in one study of general admissions to the hospital, neither bilirubin nor other liver function tests predicted 12 month mortality, while low levels of albumin did [20].

It has previously been reported that low albumin levels predict mortality in the general population [21], as well as in certain groups, such as heart failure patients [13, 22]. Low albumin levels can be seen in a number of serious conditions, such as like cancer, major infections, liver disease and inflammatory diseases, but can also indicate a poor nutritional status.

Hematocrit and iron also showed U-shaped associations with all-cause mortality in the present study. It is well known that a poor iron intake or general malabsorption can lead to anemia. Also, a poor nutritional intake of vitamin B12 or folate could also induce a low hematocrit along with a number of serious diseases, like cancer, kidney failure and inflammatory diseases. Anemia is a well-known risk factor for mortality, both in individuals with various diseases [23–25], and in the general population [23–25]; and an U-shaped relationship of hematocrit with mortality has been reported previously in smaller studies [26].

High levels of leukocytes have repeatedly been found to be related to all-cause mortality in the general population [27–29]. The underlying reasons for this may include infectious diseases or general physical and psychological stress, as reviewed by Chmielewski and Strzelec [30]. Although chemotherapy-induced leukopenia in cancer patients is associated with increased mortality [31], very little is known regarding mortality risk associated with a low leukocyte count in the general population.

One previously published study has shown both high and low levels of serum uric acid to be related to mortality in elderly individuals [32], while another investigation found an association only between high uric acid levels and mortality in a middle-aged population [33]. Serum uric acid is related to dietary intake of fat and meat [34], and therefore it is possible that low serum uric acid levels could indicate poor nutrition, which in turn is linked to mortality.

### Distribution-driven vs outcome-driven normal ranges

The use of cut-offs at the 2.5^th^ and 97.5^th^ percentile could be appropriate if an increased mortality of the same magnitude was seen both at low and high levels of the analyte. This was however not the case for most of the tests investigated in the present study. For the clinical chemistry tests related to mortality in a linear fashion, it is obvious that only a upper (or lower in the case of an inverse relationship, such as albumin) normal limit is appropriate. For U-shaped relationships, the spline regression curves were rarely symmetrical. Low levels of calcium, for example, were more strongly related to mortality than high levels, and therefore the normal range should asymmetrically defined around the median. We propose that health-driven definitions of normal ranges of commonly used clinical chemistry tests should be used rather than the distribution-driven ranges normally used today, and our data provide indications of suitable levels for such cut-offs.

### Prediction of all-cause mortality

When adding the investigated clinical chemistry tests to a model including classical risk factors to predict mortality during 10 years of follow-up, we observed a highly significant improvement in C-statistics by almost 1% in X69 or more than 2% in the UK Biobank. Although an improvement in C-statistics of 1–2% is quite substantial and more than what is typically observed in studies of clinical biomarkers, we are still uncertain whether these results could lead to a new clinically useful risk score for mortality. That said, we believe that our results highlight that these commonly used clinical chemical test include important predictive information about future pathophysiological events, not captured by the traditional risk factors. This observation further emphasizes the need for outcome-driven normal ranges, rather than distribution-driven.

We noted a substantial difference in the C-statistics (and presumably as a result, also a difference in delta C-statistics) between X69 and UK Biobank, despite a similar follow-up period (when restricting X69 to the first ten years of follow-up for comparison). Since age is the main driver of mortality prediction, we believe that the larger age range, with more young individuals, in X69 compared to UK Biobank, could be a major reason for this discrepancy in the C-statistics. Another reason is that the 10-year follow-up of X69 was in the 1970ies in Sweden, while for UK Biobank, the follow-up period was almost 40 years later in the UK. Different mortality rates during these calendar periods and geographical differences could have contributed to the different C-statistics.

### Strengths and limitations

A major strength of the present study is the unprecedented long follow-up period in large study samples providing an outstanding power to model non-linear relationships in a statistically robust fashion. Another major strength is that we could explore the associations in two independent cohorts from different countries and time periods making our observations highly robust.

Our study also had limitations. First, we performed this study in two cohorts with a majority of individuals with Western European ethnicity leading to unknown generalizability to other ethnic groups. Also, as in any observational study, our associations should be viewed as correlations and hence, useful for risk prediction and studies of normal ranges, but should not be taken to imply causality.

It can be viewed that having different follow-up periods in the different datasets and still obtain very similar results would increase the generalizability of our findings. It could also be viewed as a disadvantage with different follow-up periods, and therefore we also performed an analysis of only the first 10 years in the X69 sample and compared that to the similar follow-up time in UK biobank. Also in that case, similar results were obtained in the two samples, and therefore we used that time-frame for the prediction analyses.

The intension was to use the most general of all end-points, total mortality, to show the value of measurements of some commonly used clinical chemistry tests in a general perspective, but it can also be viewed as a limitation that we did not analyzed these clinical chemistry tests in relation to different types of cause of death. That approach might add information on pathophysiological events relating different pathways to different kinds of diseases, but was not the scope of the present study.

## Conclusion

Commonly used clinical chemistry tests were associated with all-cause mortality both in the medium- and long-term perspective, and can improve mortality prediction beyond classical risk factors. Since both linear and U-shaped relationships were found, we propose to define the normal range of a clinical chemistry test from its association with outcomes, rather than based on the distribution in the general population.
